# Performance Benefits of Pre- and Per-cooling on Self-paced Versus Constant Workload Exercise: A Systematic Review and Meta-analysis

**DOI:** 10.1007/s40279-023-01940-y

**Published:** 2023-10-06

**Authors:** Tessa M. van de Kerkhof, Coen C. W. G. Bongers, Julien D. Périard, Thijs M. H. Eijsvogels

**Affiliations:** 1 Department of Physiology (392), Radboud University Medical Center, Radboud Institute for Health Sciences, P.O. Box 9101, 6500 HB Nijmegen, The Netherlands; 2 School of Sports and Exercise, HAN University of Applied Sciences, Nijmegen, The Netherlands; 3 University of Canberra Research Institute for Sport and Exercise, Canberra, ACT Australia

## Abstract

**Background and Objective:**

Exercise in hot environments impairs endurance performance. Cooling interventions can attenuate the impact of heat stress on performance, but the influence of an exercise protocol on the magnitude of performance benefit remains unknown. This meta-analytical review compared the effects of pre- and per-cooling interventions on performance during self-paced and constant workload exercise in the heat.

**Methods:**

The study protocol was preregistered at the Open Science Framework (https://osf.io/wqjb3). A systematic literature search was performed in PubMed, Web of Science, and MEDLINE from inception to 9 June, 2023. We included studies that examined the effects of pre- or per-cooling on exercise performance in male individuals under heat stress (> 30 °C) during self-paced or constant workload exercise in cross-over design studies. Risk of bias was assessed using the Cochrane Risk of Bias Tool for randomized trials.

**Results:**

Fifty-nine studies (*n* = 563 athletes) were identified from 3300 records, of which 40 (*n* = 370 athletes) used a self-paced protocol and 19 (*n* = 193 athletes) used a constant workload protocol. Eighteen studies compared multiple cooling interventions and were included more than once (total *n* = 86 experiments and *n* = 832 paired measurements). Sixty-seven experiments used a pre-cooling intervention and 19 used a per-cooling intervention. Average ambient conditions were 34.0 °C [32.3–35.0 °C] and 50.0% [40.0–55.3%] relative humidity. Cooling interventions attenuated the performance decline in hot conditions and were more effective during a constant workload (effect size [ES] = 0.62, 95% confidence interval [CI] 0.44–0.81) compared with self-paced exercise (ES = 0.30, 95% CI 0.18–0.42, *p* = 0.004). A difference in performance outcomes between protocols was only observed with pre-cooling (ES = 0.74, 95% CI 0.50–0.98 vs ES = 0.29, 95% CI 0.17–0.42, *p* = 0.001), but not per-cooling (ES = 0.45, 95% CI 0.16–0.74 vs ES = 0.35, 95% CI 0.01–0.70, *p* = 0.68).

**Conclusions:**

Cooling interventions attenuated the decline in performance during exercise in the heat, but the magnitude of the effect is dependent on exercise protocol (self-paced vs constant workload) and cooling type (pre- vs per-cooling). Pre-cooling appears to be more effective in attenuating the decline in exercise performance during a constant workload compared with self-paced exercise protocols, whereas no differences were found in the effectiveness of per-cooling.

**Supplementary Information:**

The online version contains supplementary material available at 10.1007/s40279-023-01940-y.

## Key Points

**Table:** 

Pre-cooling is more effective in constant workload exercise compared with self-paced exercise in the heat.
Per-cooling provides comparable benefits for constant and self-paced exercise in the heat.
Substantial differences in the magnitude of performance benefits across different types of cooling interventions were observed, which emphasizes the need for more research to determine the most effective type of cooling under specific exercise conditions (e.g., type, duration).

## Introduction

Exercise in the heat results in internal heat storage, impairment of athletic performance [1], and an increased risk for heat-related illness [2, 3]. Heat mitigation strategies, such as cooling interventions and heat acclimation, have been shown to attenuate the development of thermal strain and improve exercise performance in the heat [4, 5]. Heat acclimation is regarded as the primary intervention to undertake prior to exercise in the heat [1], but requires a dedicated time frame to induce physiological adaptations. In contrast, cooling interventions can provide an immediate reduction in thermal strain by increasing heat storage capacity directly prior to exercise (pre-cooling) or attenuating the increase in core temperature during exercise (per-cooling). Cooling interventions can be applied externally (i.e., cooling garments, cold water immersion, or fanning) and internally (i.e., cold fluid or ice ingestion). Over the past decade, several reviews and meta-analyses have demonstrated that both pre-cooling and per-cooling can effectively attenuate the decline in exercise performance in the heat [6–9]. However, a limitation of previous work is that all available evidence was pooled and the type of exercise protocol (i.e., self-paced and constant workload exercise) was not factored in when evaluating the performance benefits of cooling. This may have led to over- or under-estimation of the cooling-induced performance benefits as the pooled outcomes may not be representative of exercise in a sport-specific setting (e.g., marathon running, individual time trial cycling, team sports).

Endurance performance can be assessed in laboratory settings using different protocols. The objective of self-paced exercise protocols is to complete a known distance or amount of work as quickly as possible, or maintain the highest workload for a given time, with the ability to adjust the workload based on maintaining an optimal performance intensity [1]. In contrast, constant workload protocols adopt a set work rate and individual pacing cannot occur beyond adjusting cadence or ceasing exercise. These are typically used to isolate independent variables (e.g., cooling intervention) in a well-controlled environment to examine their effect on dependent variables (e.g., volitional fatigue). Although both types of exercise protocols can reliably assess changes in exercise performance (i.e., sensitivity) [10, 11] and have external validity (i.e., representative for race conditions), the magnitude of performance change may differ markedly, with changes in time to volitional fatigue stemming from an acute intervention (e.g., heat or hypoxia) typically being much larger than those of self-paced exercise [10, 12, 13].

The aim of this systematic review and meta-analysis was to compare the effects of cooling interventions on performance outcomes during self-paced and constant workload exercise in the heat by standardizing the impact of cooling on performance and presenting effect sizes (ESs). Second, we evaluated the impact of the type of cooling (i.e., pre-cooling vs per-cooling) on exercise performance between exercise protocols. We hypothesized that cooling strategies would be equally effective between self-paced and constant workload exercise.

## Methods

### Search Strategy

This review was performed according to the Preferred Items for Systematic Reviews and Meta-Analysis—Protocol (PRISMA-P) statement [14] and was pre-registered with the Open Science Framework Registries (https://osf.io/wqjb3). A systematic literature search was conducted in PubMed, Web of Science, and MEDLINE. Three main search themes were used, which included exercise, cooling interventions, and an exercise performance outcome measure. Titles and abstracts were searched in addition to using Medical Subject Heading terms in PubMed. Words within the themes were combined using the Boolean operator “OR”, while the three themes were connected by “AND” (Table 1 of the Electronic Supplementary Material [ESM]). The final search was performed from inception up to 9 June, 2023. Search results from these databases were combined and duplicates removed using Mendeley Reference Management Software (Elsevier, London, UK). Two reviewers (T.M.K and C.C.W.G.B) screened the article titles and abstracts for inclusion; in the case of disagreement between those reviewers, a third reviewer (T.M.H.E) was consulted and decided on inclusion or exclusion. The reference list of included articles was screened for any additional articles that were missed by the literature search.

### Inclusion Criteria

Studies were included if they (1) applied a pre-cooling or a per-cooling strategy and adopted a crossover design; (2) used a self-paced or a constant workload exercise protocol; (3) were performed in hot ambient conditions (≥ 30 °C); (4) included data reported separately for male and female individuals; and (5) reported at least one outcome parameter related to exercise performance. Studies were excluded if they (1) adopted a combination of pre- and per-cooling interventions and (2) were scored with a high risk of bias [15].

### Study Classification

All included studies were classified into two groups based on the exercise protocol: self-paced or constant workload exercise. Self-paced exercise protocols were defined as exercise protocols that consisted of a fixed distance, time, or work to be completed and allowed participants to change the speed or workload during the trial. Constant workload protocols were defined as exercise protocols that were performed at a workload equivalent to a percent of maximal aerobic power (e.g., 60% of maximal oxygen consumption [*V*O_2max_]) or peak workload (e.g., 70% of peak power output), or a specified rating of perceived exertion (RPE) (e.g*.*, RPE of 15) until volitional fatigue/exhaustion. Studies adopting a warm-up and those where a pre-loaded exercise trial was performed at a different exercise protocol than the actual performance trial were classified based on the characteristics of the performance trial. For example, if a pre-loaded constant workload trial preceded a self-paced exercise trial, only the data from the self-paced trial were included in the meta-analysis.

Pre-cooling was defined as any cooling intervention applied either prior to the performance trial (i.e., at rest, warm-up, or pre-loaded trial) or during exercise breaks (e.g., 15-min half-time break). If cooling was applied both prior to the trial and during the half-time break, all data from the performance trial were used. However, if cooling was only applied during half-time, data from the second half were extracted, given that the first half was similar in both the control and intervention trials (i.e., randomized design and comparable environmental conditions). Per-cooling was defined as any cooling intervention that was applied during exercise as part of the performance trial. Studies that investigated more than one cooling intervention in separate trials were included more than once. Studies adopting multiple cooling interventions at the same time (e.g., cooling vest and cold/ice water ingestion) were classified as mixed-method cooling. We did not distinguish between or exclude non-thermal cooling methods as it has been shown to improve exercise performance [16]. We therefore also included menthol-based cooling interventions in our systematic review and meta-analysis.

### Risk of Bias Analysis

Risk of bias was assessed independently by two researchers (T.M.K and C.C.W.G.B) according to the Cochrane Risk of Bias Tool for randomized trials to assess the methodological quality of the included studies. After the initial assessment, the risk of bias of both researchers was compared and in cases where a consensus was not reached, the evaluation of a third researcher (T.M.H.E) was decisive.

### Data Extraction

Data were extracted from each study to a predefined Excel sheet (Microsoft Excel, version 16.73). This included: (1) article information (author name, year, title, study design); (2) participant characteristics (age, sex, and *V*O_2max_); (3) study characteristics (number of participants, ambient conditions [ambient temperature, relative humidity, and air flow], exercise characteristics [running, cycling], exercise protocol, exercise duration, type of cooling intervention, timing of cooling,); and (4) exercise performance data (mean ± standard deviation). For self-paced exercise, relevant outcome parameters included finish time (in seconds), total distance covered (in meters), mean power output (in Watts), total work done (in kilojoules), or peak power output (in Watts). A single measure was selected when multiple power output outcome measures were reported, prioritized as mean power output, total work done, and peak power output. For constant workload exercise, outcome measures included time to exhaustion (in seconds), mean power output (in Watts), total work done (in kilojoules), or peak power output (in Watts). A single measure was selected when multiple power output outcome measures were reported, prioritized as mean power output, total work done and peak power output. In the case of missing data, only the available data were analyzed and presented. In case data were not explicitly provided in the text, but only in a figure, data were extracted using a validated graphical software program (WebPlotDigitizer version 4.5; Automeris LLC, Pacifica, CA, USA) by a single experienced researcher [17, 18].

### Data Synthesis and Analysis

Data analysis was performed on raw data (means, standard deviation, and sample size) using Review Manager (version 5.4), in line with the Cochrane guidelines. For all included studies, the standardized mean difference was calculated as the Hedges’ ES (ES = difference in outcome between conditions/standard deviation of outcome among participants) with a corresponding 95% confidence interval (CI) [19]. The magnitude of Hedges’ *g* was interpreted as: < 0.2, trivial; 0.2–0.49, small effect; 0.5–0.79, moderate effect; ≥ 0.8, large effect [20]. Heterogeneity was assessed using I^2^ statistics with < 25% being considered low heterogeneity and > 75% high heterogeneity [21]. A fixed-effects model was used to calculate the pooled weighted average ES to correct for differences in the sample size between studies by using the inverse-variance weighted average method [22]. Stratified analyses were also performed to compare the effect of cooling type (pre- vs per-cooling) between self-paced and constant workload exercise protocols. Exploratory analyses were additionally performed to assess the impact of exercise duration on the ES. Potential publication bias was assessed by visual inspection of funnel plot asymmetry. All data were presented as mean ± standard deviation. To assess between-study normality of data, a Kolmogorov–Smirnov test was performed; in the case of non-normality, the median with interquartile range was reported. The significance level for all statistical tests was set at *p* < 0.05. Data analyses on the ESs were conducted using Review Manager, whereas publication bias was assessed using Rstudio (version 1.4.1106; packages: tidyverse, meta, metafor).

## Results

### Participants and Included Studies

**Fig. 1:**
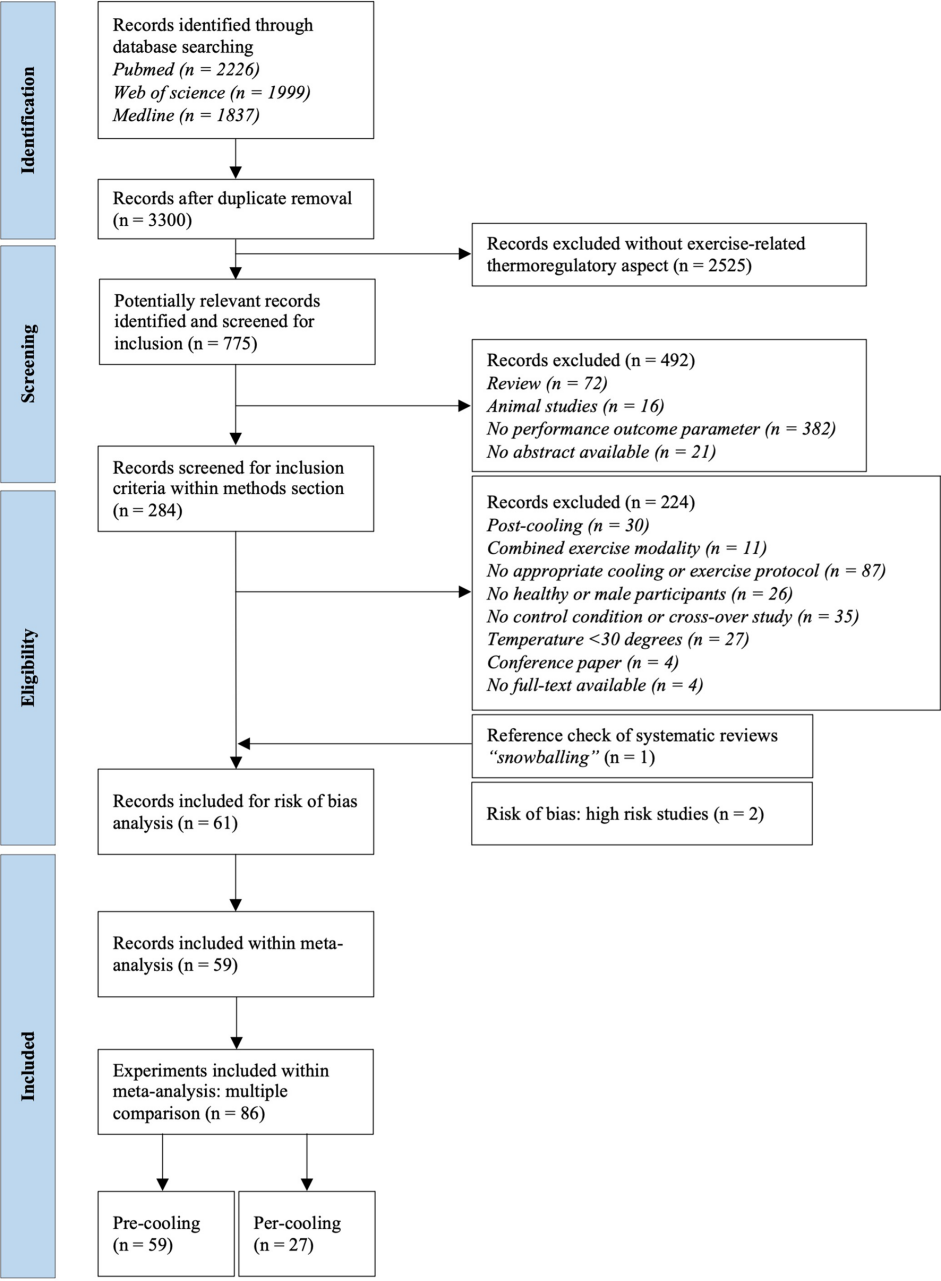
Flow chart of the systematic search and study selection process

The literature search identified 3300 articles after the removal of duplicates. After the initial title and abstract screening and subsequent full-text screening, 61 studies complied with our inclusion criteria, of which two [23, 24] were excluded owing to a high risk of bias because of missing data (Fig. 1). In total, 59 studies (*n* = 563 athletes, age: 24.0 [21.0–26.0] years, *V*O_2peak_: 55.8 ± 6.0 mL kg^−1^ min^−1^) were included in the meta-analysis, of which 40 studies (*n* = 370 athletes) comprised a self-paced exercise protocol [25–64] (Table 1) and 19 studies (*n* = 193 athletes) comprised a constant workload protocol [65–83] (Table 2). A total of 18 studies compared multiple cooling interventions and were therefore included more than once. This resulted in 86 experiments (*n* = 832 paired measurements) in which exercise performance was compared between the control and cooling conditions. Almost all studies were conducted in an indoor laboratory setting (*n* = 56; 95%). Average ambient conditions were 34.0 °C [32.3–35.0 °C] and 50.0% [40.0–55.3%] relative humidity and did not differ between self-paced and constant workload studies (*p* = 0.11 and *p* = 0.49, respectively). Furthermore, 22 out of 59 studies (37%) reported information on airflow, which was 2.1 ± 1.5 m s^−1^ on average and did not differ between study protocols (*p* = 0.40).

**Table 1:** Study characteristics, self-paced studies

Study	Age (years)	*V*O_2max_ (mL kg^−1^ min^−1^) or fitness level	Type of exercise (primary outcome measure)	Type of cooling	Method of cooling	Ambient conditions	Airflow	Lab/field study
*Pre-cooling*								
Arngrimmson et al. 2004 (*n* = 9) [25]	23 ± 4	66.7 ± 5.9	5-km running time trial (time)	Pre-cooling	Cooling vest during warm-up	32 °C/50% RH	Unknown	Lab, indoor
Brade et al. 2014 (*n* = 12) [26]	22 ± 2	Team sport players	2 × 30-min repeat sprint cycling, 10-min half-time (mean power output)	Pre-cooling	(a) PCM cooling vest during rest + half-time (b) Ice slushy (7 g/kg BM) during rest; 2.1 g/kg BM during half-time (c) Cooling vest and ice slushy during rest + half-time	35 °C/60% RH	Unknown	Lab, indoor
Byrne et al. 2011 (*n* = 7) [37]	21 ± 2	Recreational cyclists	30-min cycling time trial (total distance covered)	Pre-cooling	Cold water ingestion (2 °C, 900 mL) during rest	32 °C/60% RH	3.2 m/s (fan)	Lab, indoor
Castle et al. 2006 (*n* = 12) [48]	23 ± 1	47.3 ± 2.4	20 × 2-min intermittent cycling sprint protocol (total work done)	Pre-cooling	(a) Cooling vest (10.7 °C) during rest (b) Cold water immersion (17.8 °C) during rest (c) Cooling packs (− 16.0 °C) during rest	33.7 °C/51.6% RH	Unknown	Lab, indoor
Chaen et al. 2019 (*n* = 8) [59]	21 ± 2	Well trained	2 × 30-min intermittent cycling protocol, 15-min half-time (mean power output)	Pre-cooling	Cooling vest (− 1 °C) during half-time	33 °C/50% RH	Unknown	Lab, indoor
Coelho et al. 2021 (*n* = 15) [60]	23 ± 4	42.3 ± 4.4	5-km running time trial (time)	Pre-cooling	Head cooling (− 20.2 °C) during rest	35 °C/50% RH	1.5 m/s	Lab, indoor
Duffield et al. 2003 (*n* = 7) [61]	20 ± 2	Well trained	4 × 15-min intermittent cycling sprint protocol, 10-min half-time (mean power output)	Pre-cooling	Cooling vest during rest + each break	30 °C/60% RH	Unknown	Lab, indoor
Duffield et al. 2007 (*n* = 9) [62]	21 ± 1	Well trained	2 × 30-min intermittent running sprint protocol, 10-min half-time (total distance covered)	Pre-cooling	(a) Cold water immersion (14 °C) during rest and cooling vest during warm-up + half-time (b) Cooling vest during rest + warm-up + half-time	32 °C/30% RH	Unknown	Lab, indoor
Duffield et al. 2009 (*n* = 7) [63]	20 ± 1	Well trained	4 × 5-min intermittent running sprint protocol, 10-min half-time (total distance covered)	Pre-cooling	Cooling vest and cooling packs during rest	32.4 °C/44.0% RH	Unknown	Lab, indoor
Duffield et al. 2010 (*n* = 8) [64]	25 ± 3	Well trained	40-min cycling time trial (mean power output)	Pre-cooling	Cold water immersion (14 °C) during rest	33 °C/50% RH	No additional airflow	Lab, indoor
Duffield et al. 2013 (*n* = 9) [27]	23 ± 3	Well trained	2 × 10-min intermittent running and 6 × 3-min small-sided games, 5-min recovery (total distance covered)	Pre-cooling	Cooling vest during rest + half-time and cold towel (5 °C) during rest + half-time and ice slurry ingestion (350 mL) during rest	30 °C/75% RH	Unknown	Field, outdoor
Faulkner et al. 2015 (*n* = 10) [28]	25 ± 6	61.3 ± 4.3	75% W_max_ cycling time trial (time)	Pre-cooling	(a) Evaporative and conductive cooling vest (COLD) during rest (b) Evaporative cooling vest (COOL; 14.3 °C) during rest	35.0 °C/50.6% RH	Unknown	Lab, indoor
Faulkner et al. 2019 (*n* = 8) [29]	25 ± 6	61.3 ± 4.3	75% W_max_ cycling time trial (time)	Pre-cooling	Evaporative and conductive cooling vest (COLD) during rest	35.0 °C/50.6% RH	3 m/s (fan)	Lab, indoor
Fiol et al. 2021 (*n* = 12) [30]	26 ± 4	57.6 ± 7.9	5-km cycling time trial (time)	Pre-cooling	(a) Low-dose cold air inhalation (60 s on; 4 min off) during preloaded trial (b) High-dose cold air inhalation (60 s on; 60 s off) during preloaded trial	30 °C/55% RH	3.5 m/s (fan)	Lab, indoor
Gerrett et al. 2017 (*n* = 12) [31]	30 ± 3	58.5 ± 8.1	31-min intermittent running protocol (total distance covered)	Pre-cooling	Ice slurry ingestion (7.5 g/kg; 0.14 °C) during rest	30.2 °C/42.5% RH	1.3 m/s (fan)	Lab, indoor
Ihsan et al. 2010 (*n* = 7) [32]	28 ± 3	Trained	40-km cycling time trial (time)	Pre-cooling	Ice slurry ingestion (6.8 g/kg BM; 1.4 °C) during rest	30 °C/75% RH	Unknown	Lab, indoor
Katica et al. 2018 (*n* = 8) [33]	25 ± 3	50.2 ± 7.2	16.1-km cycling time trial (time)	Pre-cooling	Cooling vest during warm-up	35.0 °C/43.8% RH	3.3 m/s	Lab, indoor
Kay et al. 1999 (*n* = 8) [34]	24 ± 2	64.5 ± 3.3	30-min cycling time trial (total distance covered)	Pre-cooling	Cold water immersion (8–11 °C) during rest	31.4 °C/60.2% RH	Unknown	Lab, indoor
Maia-Lima et al. 2017 (*n* = 8) [35]	28 ± 3	55.7 ± 7.9	30-km cycling time trial (time)	Pre-cooling	Cold water immersion (24 °C) during rest	35 °C/68% RH	0.5 m/s (fan)	Lab, indoor
Maroni et al. 2018 (*n* = 12) [36]	21 ± 2	Trained	2 × 30-min repeat sprint cycling protocol, 10-min half-time (mean power output)	Pre-cooling	(a) Cooling glove (~ 16 °C) during half-time (b) Cooling jacket (0–2 °C) during half-time (c) Cooling glove (~ 16 °C) and cooling jacket (0–2 °C) during half-time	35.0 °C/52.5% RH	No active airflow	Lab, indoor
Maroni et al. 2020 (*n* = 10) [38]	21 ± 3	65.7 ± 10.7	6 × 15-s cycling sprint with varying recovery times + 2 × 5-min time trial (mean power output)	Pre-cooling	(a) Cooling glove during rest + warm-up (b) Cooling jacket (0–2 °C) during rest + warm-up (c) Cooling glove and cooling jacket (0–2 °C) during rest + warm-up	35.0 °C/56.6% RH	Unknown	Lab, indoor
Mazalan et al. 2022 (*n* = 9) [39]	28 ± 3	Trained	30-min intermittent cycling sprint protocol (total work done)	Pre-cooling	Ice slurry ingestion (7 g/kg; − 0.4 °C)	35.0 °C/70% RH	Unknown	Lab, indoor
Minett et al. 2011 (*n* = 10) [40]	21 ± 3	Well trained	2 × 35-min intermittent running sprint protocol (total distance covered)	Pre-cooling	(a) Head cooling (5.0 °C) during rest + half-time (b) Head cooling (5.0 °C) and hand immersion (9.0 °C) during rest + half-time (c) Mixed-method whole body cooling (head cooling and hand immersion and cooling vest and cooling packs) during rest + half-time	33.0 °C/33.3% RH	Unknown	Lab, indoor
Minett et al. 2012a (*n* = 10) [41]	23 ± 8	Well trained	45-min 6-over bowling spell	Precooling	Wet towel (5.0 °C) on head, neck, and shoulders and cooling vest (− 20 °C) and wrist immersion (9.0 °C) and cooling packs (− 20 °C) on legs during rest	31.9 °C/63.5% RH	Unknown	Field, outdoor
Minett et al. 2012b (*n* = 8) [42]	22 ± 3	Well trained	2 × 35-min intermittent running protocol (total distance covered)	Pre-cooling	Head cooling (5.0 °C) and hand immersion (9.0 °C) and cooling vest and cooling packs during rest + half-time	33.0 °C/33.9% RH	Unknown	Lab, indoor
Moss et al. 2021 (*n* = 9) [43]	32 ± 10	65 ± 7	15-min cycling time trial (total distance covered)	Pre-cooling	(a) Cold water immersion (22–24 °C) and cold water ingestion (1.25 mL/kg; ~ 10 °C) during rest (b) Cooling collar during pre-loaded trial (c) Cold water immersion (22–24 °C) and cold water ingestion (1.25 mL/kg; ~ 10 °C) during rest and cooling collar during pre-loaded trial	40 °C/50% RH	1.5 m/s	Lab, indoor
Naito et al. 2020 (*n* = 7) [44]	31 ± 4	Physically active	30 × 1-min sprint cycling protocol (mean power output)	Pre-cooling	Ice slurry ingestion (1.25 mL/kg BM; − 1 °C) during each break and half-time	36.5 °C/50% RH	Unknown	Lab, indoor
Skein et al. 2012 (*n* = 10) [45]	20 ± 1	Physically active	50-min intermittent running protocol (total distance covered)	Pre-cooling	Cold water immersion (10 °C) during rest	31 °C/33% RH	Unknown	Lab, indoor
Stevens et al. 2016 (*n* = 11) [46]	29 ± 9	Moderately trained	5-km running time trial (time)	Pre-cooling	Ice slurry ingestion (7.5 g/kg BM; − 1 °C) during rest	33 °C/46% RH	4 m/s (fan)	Lab, indoor
Stevens et al. 2017a (*n* = 11) [47]	30 ± 9	61 ± 6	3-km running time trial (time)	Pre-cooling	Cold water immersion (23–24 °C) and ice slurry ingestion (7.5 g/kg BM) during rest	32.5 °C/46.8% RH	4 m/s (fan)	Lab, indoor
Stevens et al. 2017b (*n* = 9) [49]	30 ± 12	Trained	5-km running time trial (time)	Pre-cooling	Cold water immersion (23–24 °C) during rest	33 °C/34% RH	4 m/s (fan)	Lab, indoor
Thomas et al. 2019 (*n* = 10) [50]	31 ± 6	56.2 ± 6.6	46-min intermittent running protocol (total distance covered)	Pre-cooling	(a) Ice slurry ingestion (7.5 g/kg BM; − 0.5 °C) during rest (b) Cooling vest (23.4 °C during rest (c) Ice slurry ingestion (7.5 g/kg BM; − 0.5 °C) and cooling vest (23.4 °C) during rest	34.4 °C/36.3% RH	1.3 m/s (fan)	Lab, indoor
Yanaoka et al. 2022 (*n* = 9) [51]	21 ± 2	57.2 ± 5.4	5-min cycling time trial	Pre-cooling	Ice slurry ingestion (5.0 g/kg; − 1.3 °C) and cooling vest during 15-min break	35 °C/50% RH	No additional airflow	Lab, indoor
Zhang et al. 2022 (*n* = 11) [52]	21 ± 2	Physciall active	Yo-Yo intermittent running protocol (total distance covered)	Pre-cooling	(a) Lower limb cold water immersion (15 °C) during 15-min break (b) Whole body cold water immersion (15 °C) during 15-min break	39.7 °C/unknown	Unknown	Field, outdoor
*Per-cooling*								
Barwood et al. 2015 (*n* = 8) [53]	21 ± 2	Physically active	16.1-km cycling time trial (time)	Per-cooling	Menthol spray (100 mL) at 10th km of time trial	33.5 °C/33% RH	2.25 m/s (fan)	Lab, indoor
Carvalho et al. 2014 (*n* = 10) [54]	25 ± 1	67.2 ± 1.8	40-km cycling time trial (time)	Per-cooling	Cold water ingestion (10 °C) during time trial	35 °C/60% RH	0.5 m/s	Lab, indoor
Hsu et al. 2005 (*n* = 8) [55]	27 ± 2	54.1 ± 3.1	30-km cycling time trial (time)	Per-cooling	Hand cooling (22 °C) during time trial	31.9 °C/24% RH	No additional airflow	Lab, indoor
Minniti et al. 2011 (*n* = 8) [56]	25 ± 5	53.7 ± 4.7	15-min running time trial (total distance covered)	Per-cooling	Neck cooling collar during preloaded trial + time trial	30.4 °C/53% RH	Unknown	Lab, indoor
Stevens et al. 2016 (*n* = 11) [46]	29 ± 9	Moderately trained	5-km running time trial (time)	Per-cooling	Menthol swilling (25 mL; 22 °C) at the 0.2-km mark of every 1 km	33 °C/46% RH	4 m/s (fan)	Lab, indoor
Stevens et al. 2017b (*n* = 9) [49]	30 ± 12	Trained	5-km running time trial (time)	Per-cooling	Facial water spray (3 sprays; 22 °C) at the 0.2-km mark of every 1 km	33 °C/34% RH	4 m/s (fan)	Lab, indoor
Sunderland et al. 2015 (*n* = 7) [57]	26 ± 3	53.5 ± 2.7	5 × 6-s sprints and 2 × 45-min football specific intermittent protocol (mean power output), 15-min half-time	Per-cooling	Neck cooling collar during whole trial	33.0 °C/53% RH	Unknown	Lab, indoor
Tyler et al. 2010 (*n* = 8) [58]	25 ± 3	54.9 ± 3.1	15-min time trial (total distance covered)	Per-cooling	Neck cooling collar during whole trial	30.4 °C/53% RH	Unknown	Lab, indoor

*BM* body mass, *lab* laboratory, *min* minutes, *PCM* phase change material, *RH* relative humidity, *sec* seconds, *VO*_*2max*_ maximal oxygen consumption, *W*_*max*_ maximal workload

**Table 2:** Study characteristics, constant workload studies

Study	Age (years)	VO_2max_ (mL kg^−1^ min^−1^) or fitness level	Type of exercise (primary outcome measure)	Type of cooling	Method of cooling	Ambient conditions	Airflow	Lab/field study
*Pre-cooling*								
Barwood et al. 2019 (*n* = 8) [65]	22 ± 2	Trained	Cycling test to exhaustion at 70% P_max_, (time to exhaustion)	Pre-cooling	Menthol spray (100 mL) during pre-loaded trial	35 °C/20% RH	1.85 m/s (fan)	Lab, indoor
Choo et al. 2019 (*n* = 11) [66]	30 ± 6	51.1 ± 8.2	60-min cycling trial at 15 RPE/’hard or heavy’ (mean power output)	Pre-cooling	(a) Ice slurry ingestion (1.25 g/kg/5 min; 0.1 °C) during rest (b) Cold water immersion (22.3 °C) during rest	33.9 °C/42.5% RH	Unknown	Lab, indoor
Hasegawa et al. 2005 (*n* = 9) [76]	22 ± 1	48.3 ± 1.7	Cycling test to exhaustion at 80% VO_2,max_ (time to exhaustion)	Pre-cooling	Cooling jacket during pre-loaded trial	32.0 °C/70–80% RH	Unknown	Lab, indoor
Mitchell et al. 2003 (*n* = 11) [77]	24 ± 7	54.8 ± 4.2	Running test to exhaustion at 100% VO_2,max_ (time to exhaustion)	Pre-cooling	Fan cooling with water spray (100 mL/2 min) during rest	38 °C/40% RH	4.0 m/s (fan)	Lab, indoor
Nakamura et al. 2020 (*n* = 8) [78]	22 ± 1	42.4	Cycling text to exhaustion at 75% VO_2,max_ (time to exhaustion)	Pre-cooling	a) Hand and forearm immersion in cold water (10 °C) during rest b) Ice slurry ingestion (4 g/kg BM; − 1 °C) during rest c) Hand and forearm immersion in cold water (10 °C) and ice slurry ingestion (4 g/kg BM; − 1 °C)	35.0 °C/62.5% RH	Unknown	Lab, indoor
Osakabe et al. 2021 (*n* = 8) [79]	23 ± 1	51.8 ± 5.0	2 × 30-min intermittent cycling protocol, 15-min half-time (mean power output)	Pre-cooling	Fan with skin wetting (~ 20 °C) during half-time	35 °C/50% RH	0.8 m/s (fan)	Lab, indoor
Siegel et al. 2012 (*n* = 8) [80]	26 ± 4	54.2 ± 2.5	Running to exhaustion at first ventilatory threshold (time to exhaustion	Pre-cooling	(a) Ice slurry ingestion (7.5 g/kg; − 1 °C) during rest (b) Cold water immersion (24 °C) during rest	34.0 °C/52% RH	Unknown	Lab, indoor
Uckert et al. 2007 (*n* = 20) [81]	26 ± 4	Trained	Running incremental step test to exhaustion (time to exhaustion)	Pre-cooling	Cooling vest (0–5 °C) during rest	30–32 °C/50% RH	Unknown	Lab, indoor
Walters et al. 2017 (*n* = 22) [82]	20 ± 2	Recreationally active	Cycling graded exercise test	Pre-cooling	Cooling cap (5–10 °C) during rest	35 °C/15% RH	Unknown	Lab, indoor
Xu et al. 2021 (*n* = 7) [83]	20 ± 1	60.7 ± 4.1	Running to exhaustion at 80% VO_2,max_ (time to exhaustion)	Pre-cooling	(a) Cooling vest (4 °C) during rest (b) Cold fluid ingestion (2.3 mL/kg BM; 4 °C) during rest (c) Cooling vest (4 °C) and cold fluid ingestion (2.3 mL/kg BM; 4 °C) during rest	38.1 °C/55.3% RH	Unknown	Lab, indoor
*Per-cooling*								
Cuttel et al. 2016 (*n* = 8) [67]	24 ± 4	Recreationally active	Cycling to exhaustion at 60% W_max_ (time to exhaustion)	Per-cooling	(a) Cooling vest (stored at − 24 °C) during whole trial (b) Neck cooling collar (stored at − 24 °C) during time trial	35 °C/50.1% RH	Unknown	Lab, indoor
Flood et al. 2017 (*n* = 8) [68]	26 ± 5	55.4 ± 6.0	Cycling to exhaustion at 16 RPE/’hard or very hard’ (time to exhaustion)	Per-cooling	Menthol mouth rinse (25 mL every 10 min; 19.8 °C) during whole trial	35.0 °C/47.8% RH	Unknown	Lab, indoor
Jeffries et al. 2018 (*n* = 10) [69]	33 ± 9	52.4 ± 5.3	Cycling to exhaustion at 70% W_max_ (time to exhaustion)	Per-cooling	(a) Ice slurry ingestion (1.25 g/kg; 0.3 °C) at 85% of baseline time to exhaustion (b) Menthol mouth rinse (25 mL; 19.5 °C) at 85% of baseline time to exhaustion	35 °C/40% RH	Unknown	Lab, indoor
Luomala et al. 2012 (*n* = 7) [70]	32 ± 3	56 ± 3	Cycling in 10-min cycles until exhaustion; 9 min at 60% VO_2,max_, 1 min at 80% VO_2,max_ (time to exhaustion)	Per-cooling	Cooling vest (− 20 °C) from 30 min into exercise until the end	30 °C/40% RH	Unknown	Lab, indoor
Mündel et al. 2006 (*n* = 8) [71]	26 ± 7	54 ± 5	Cycling to exhaustion at 65% W_max_ (time to exhaustion)	Per-cooling	Cold drink ingestion (≥ 300 mL; 3.6° C) during whole trial	33.9 °C/27.9% RH	0.5 m/s (fan)	Lab, indoor
Mündel and Jones 2010 (*n* = 9) [72]	25 ± 7	54 ± 5	Cycling to exhaustion at 65% W_max_ (time to exhaustion)	Per-cooling	Menthol mouth rinse (25 mL every 10 min; 19 °C) during whole trial	34 °C/27% RH	0.5 m/s (fan)	Lab, indoor
Parton et al. 2020 (*n* = 11) [73]	20 ± 1	53.9 ± 6.9	Cycling to exhaustion at 16 RPE/’hard or very hard’ (time to exhaustion)	Per-cooling	Menthol mouth rinse (25 mL every 10 min; 31.8 °C) during whole trial	34.9 °C/40.6% RH	Unknown	Lab, indoor
Scheadler et al. 2013 (*n* = 12) [74]	23 ± 4	53.8 ± 5.2	Running to exhaustion at 75% VO_2,max_ (time to exhaustion)	Per-cooling	Palm cooling device during whole trial	30 °C/50% RH	Unknown	Lab, indoor
Tyler et al. 2011 (*n* = 8) [75]	26 ± 2	56.2 ± 9.2	Running to exhaustion at 70% VO_2,max_ (time to exhaustion)	Per-cooling	Neck cooling collar (stored at − 80 °C for 24–28 h, 10 min in ambient conditions) during whole trial	32.2 °C/53% RH	Unknown	Lab, indoor

*BM *body mass, *lab* laboratory, *min* minutes, *PCM phase change material*, *RH* relative humidity, *RPE* rate of perceived exertion, *VO*_*2max*_ maximal oxygen consumption, *W*_*max *maximal workload_

### Risk of Bias Analysis

**Fig. 2:**
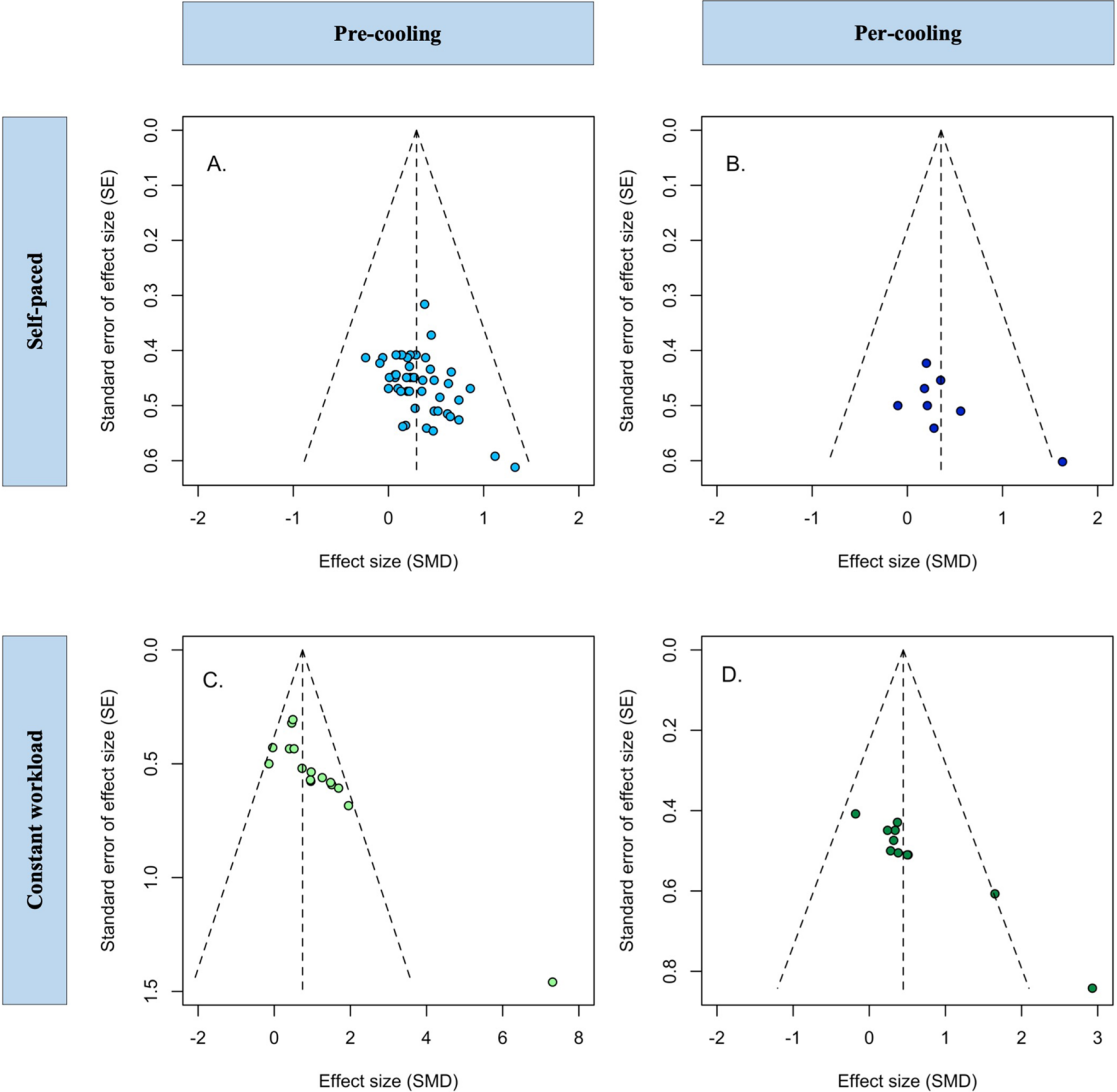
Funnel plot of included studies separated for self-paced (top figures; **A** and** B**) and constant workload (bottom figures; **C** and **D**) exercise performance; data are also separated for pre-cooling (left figures; **A** and **C**) and per-cooling (right figures; **B** and **D**). A few outliers are observed within figures **B**, **C**, and **D**. The vertical dotted line represents the weighted average effect size of all included studies. *SE* standard error, *SMD* standardized mean difference

A few outliers and little asymmetry were observed in the funnel plots for the self-paced and constant workload exercise protocol studies (Fig. 2). The risk of bias analysis revealed that 98% of included studies had “some concerns” (Tables 3, 4). This mainly related to missing information on the concealment of the allocation sequence until participants were assigned to an intervention (i.e., Domain 1) as well as missing information on whether a pre-specified analysis plan was used or not (i.e., Domain 5). The risk of bias was comparable between the self-paced and constant workload exercise protocol studies.

**Table 3:**
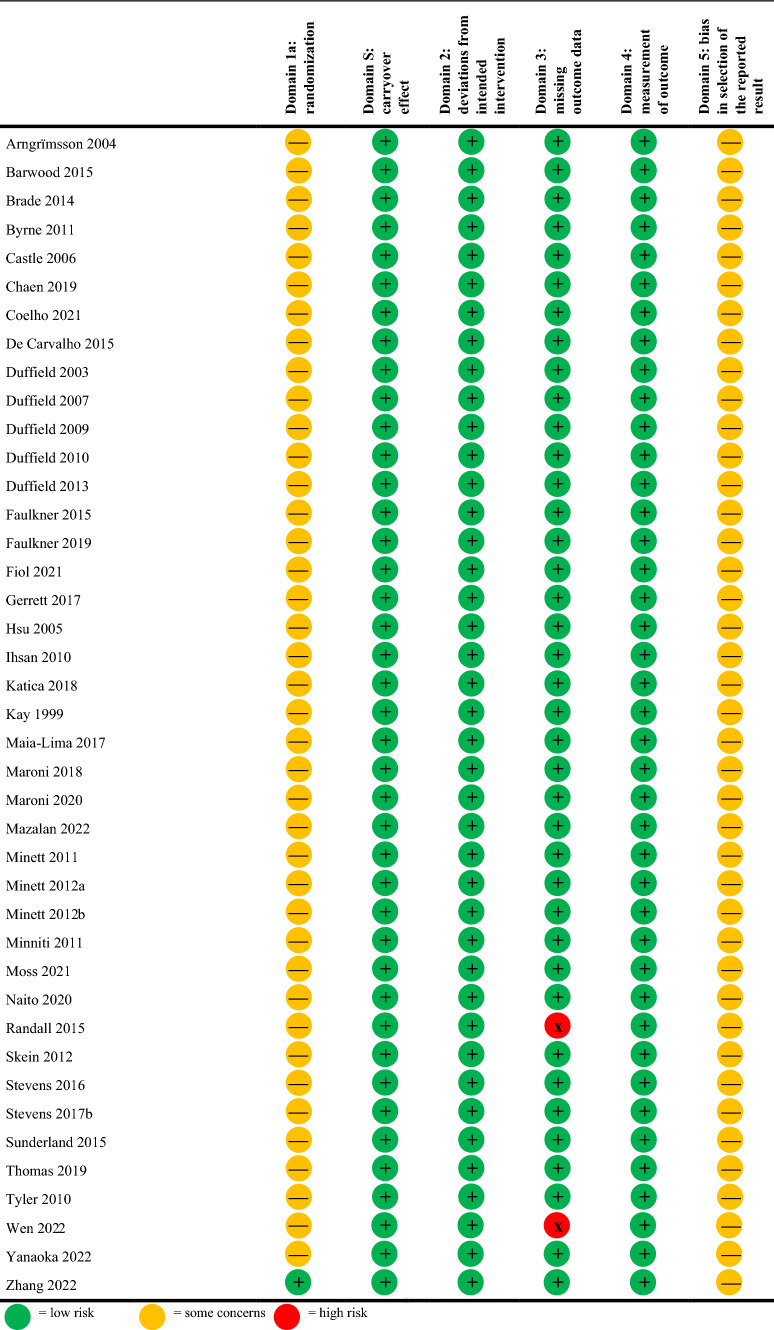
Risk of bias, self-paced studies

**Table 4:**
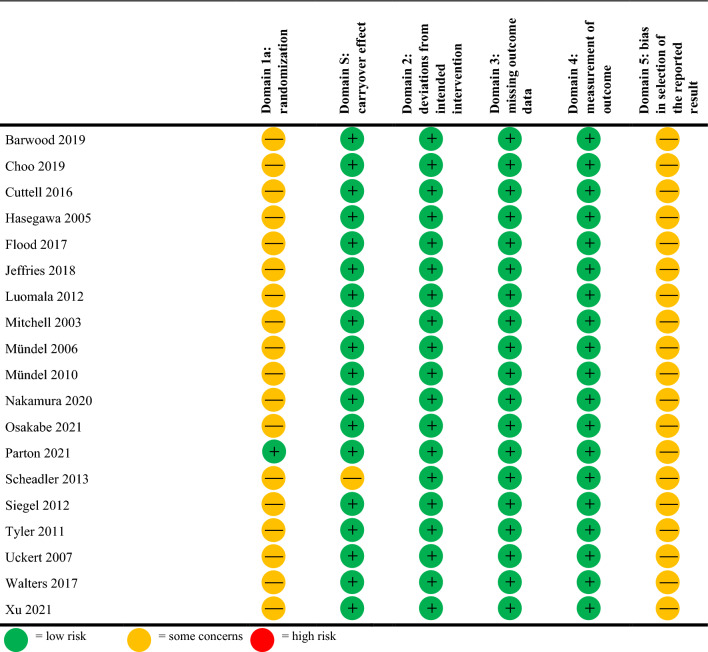
Risk of bias, constant workload studies

### Self-Paced Exercise Studies

Fifty-nine experiments (exercise duration: 40.0 [27.0–60.0] minutes) were available for self-paced exercise performance analysis, of which 51 used a pre-cooling intervention and eight used a per-cooling intervention. Mixed-method cooling (25.0%), cooling vests (18.7%), and cold/ice water ingestion (14.9%) were most frequently adopted as cooling strategies (Fig. 1 of the ESM). The median weighted improvement in self-paced exercise performance corresponded to an ES = 0.30, 95% CI 0.18–0.42.

**Fig. 3:**
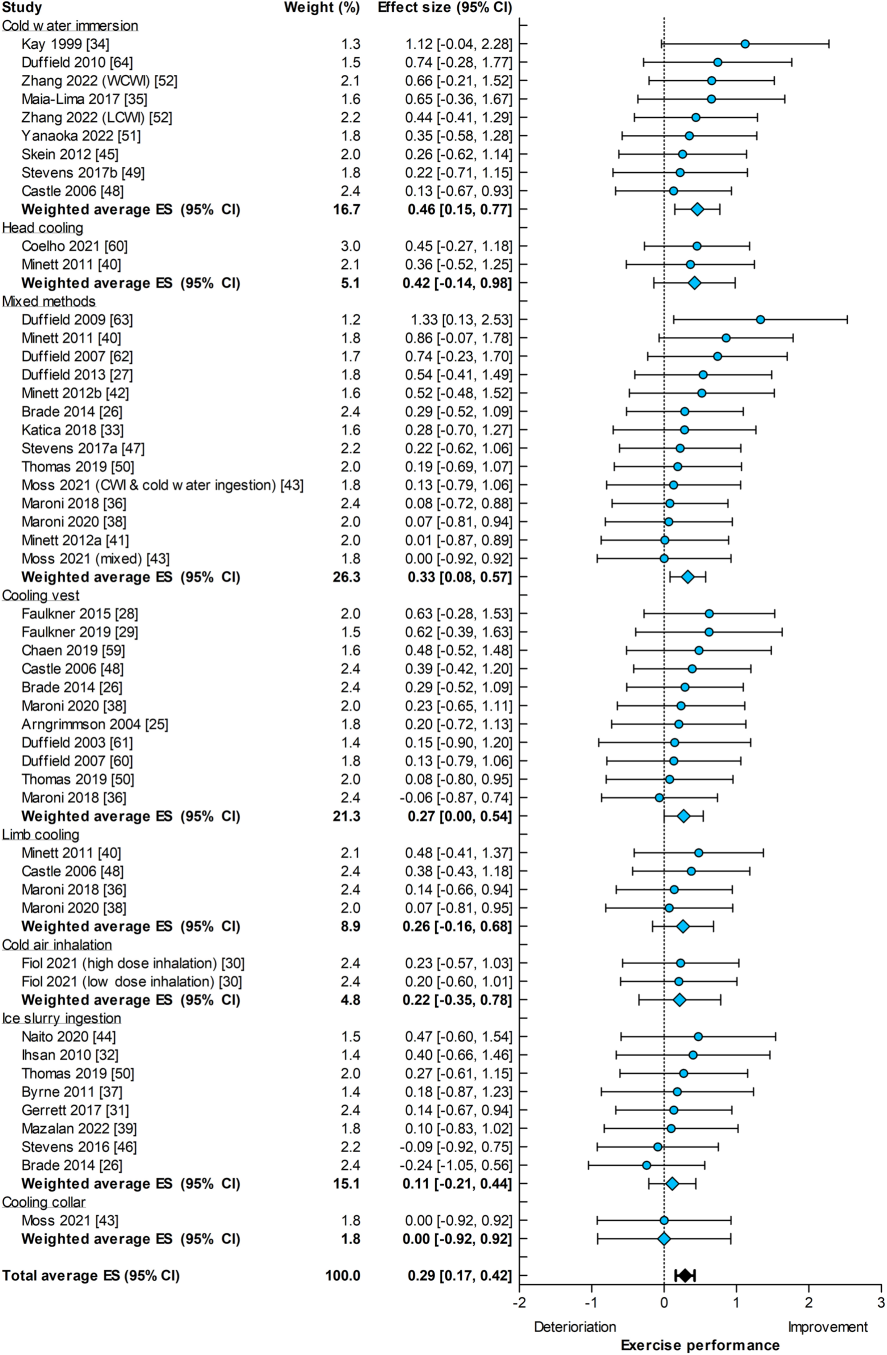
Forest plot summarizing the effects of pre-cooling on self-paced exercise performance (effect size [ES] in Hedges’ *g*), stratified for cooling interventions and sorted by effect size. The dots represent the ES; the diamonds represent the weighted average ES; the *error bars* indicate the 95% confidence interval (CI). Studies that used multiple cooling trials were included more than once. *CWI* cold water immersion, *LCWI *lower limb cold water immersion, *WCWI* whole-body cold water immersion

**Fig. 4:**
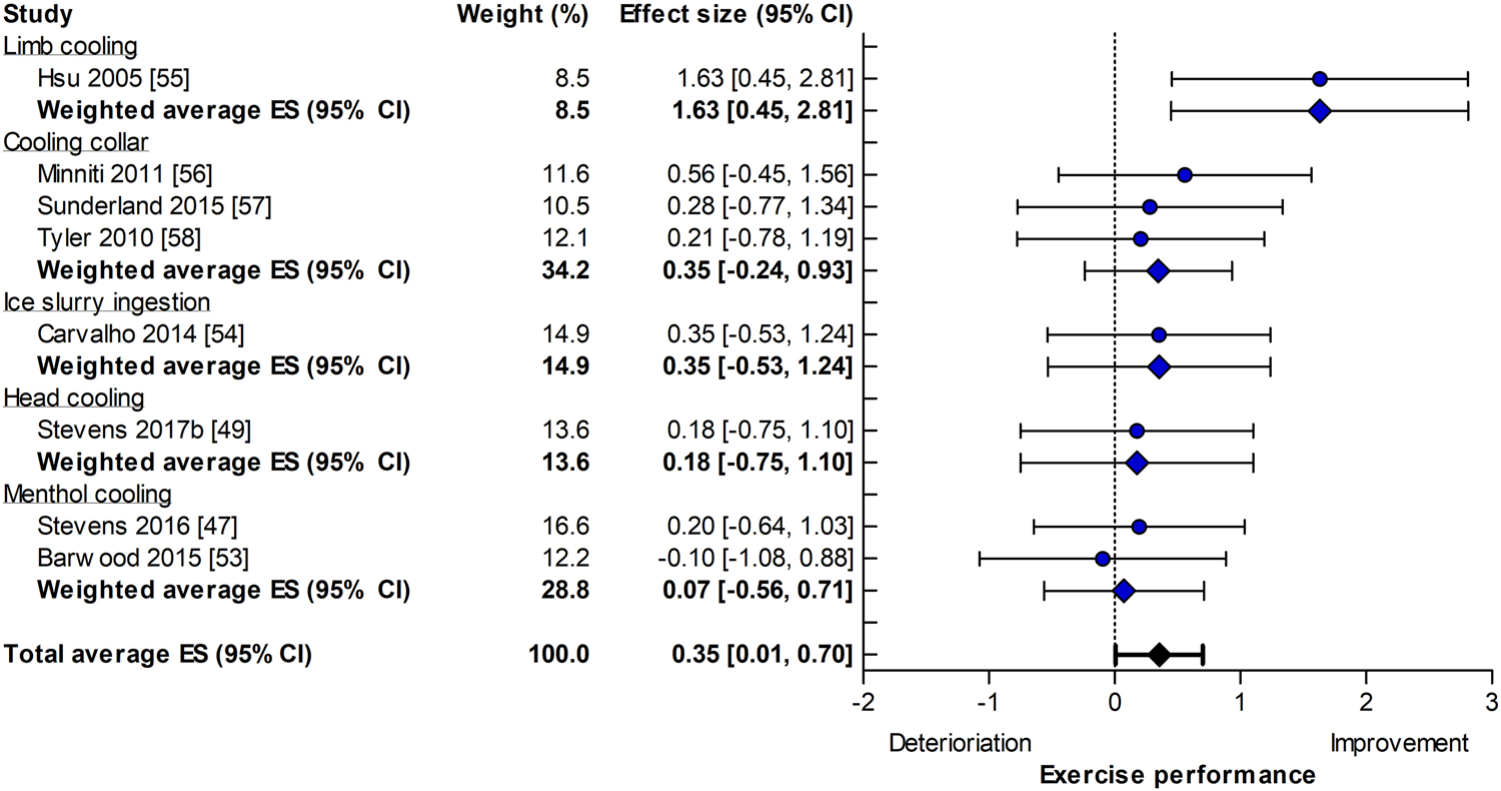
Forest plot summarizing the effects of per-cooling on self-paced exercise performance (effect size [ES] in Hedges’ *g*), stratified for cooling interventions and sorted by ES. The *dots* represent the ES; the diamonds represent the weighted average ES; the error bars indicate the 95% confidence interval (CI). Studies that used multiple cooling trials were included more than once

Pre-cooling was applied prior to a time trial (19 out of 51 experiments) or an intermittent sprint protocol (32 out of 51 experiments), whereas per-cooling was predominantly used during a time trial (seven out of eight experiments). The improvement in self-paced exercise performance was similar for pre-cooling (ES = 0.29, 95% CI 0.17–0.42, Fig. 3) and per-cooling (ES = 0.35, 95% CI 0.01–0.70, Fig. 4, *p* = 0.74). We also observed a large variability in the magnitude of the ES across cooling strategies, with no benefits from a cooling collar (ES = 0.00, 95% CI − 0.92 to 0.92) or small benefits from cold water immersion (ES = 0.47, 95% CI 0.15–0.80) for pre-cooling studies (Fig. 3), to large effects using limb cooling (ES = 1.63, 95% CI 0.45–2.81) as a per-cooling intervention (Fig. 4). Finally, no statistical heterogeneity was observed for pre- and per-cooling subgroups (*I*^2^ = 0%, *p* = 1.00 and *I*^2^ = 0%, *p* = 0.55, respectively).

### Constant Workload Exercise Studies

**Fig. 5:**
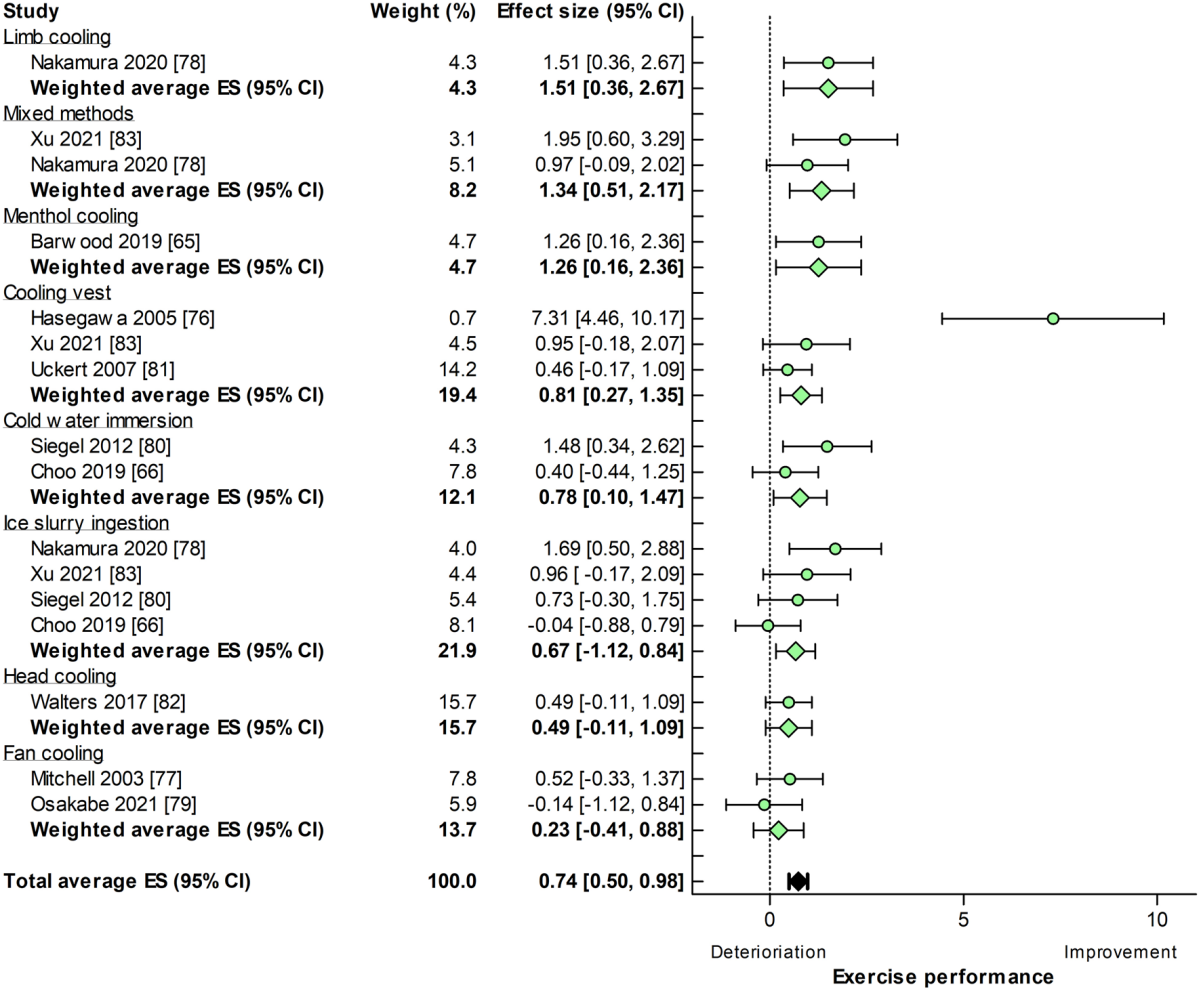
Forest plot summarizing the effects of pre-cooling on constant workload exercise performance (effect size [ES] in Hedges’ *g*), stratified for cooling interventions and sorted by effect size. The dots represent the ES; the diamonds represent the weighted average ES; the error bars indicate the 95% confidence interval (CI). Studies that used multiple cooling trials were included more than once

**Fig. 6:**
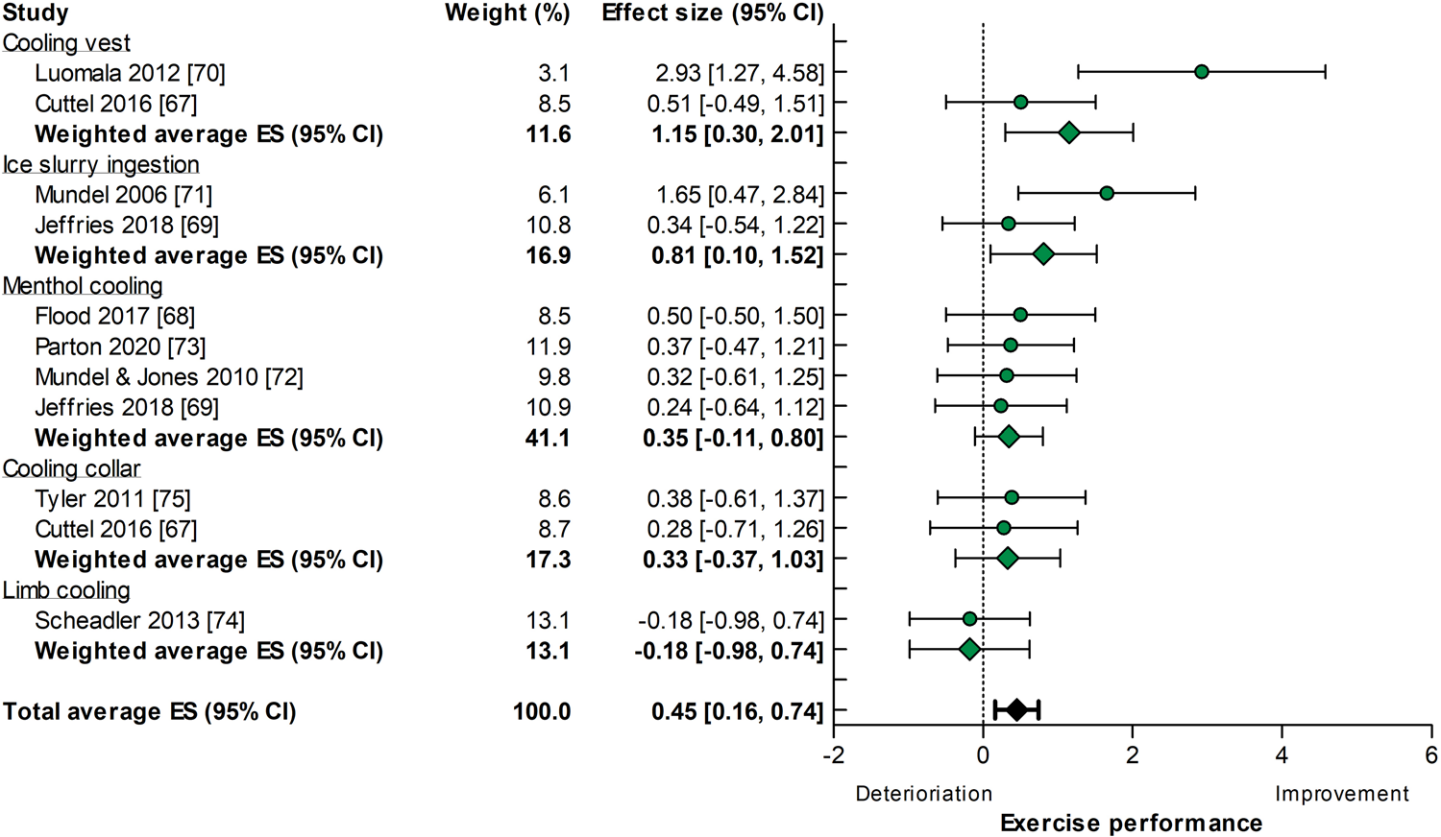
Forest plot summarizing the effects of per-cooling on constant workload exercise performance (effect size [ES] in Hedges’ *g*), stratified for cooling interventions and sorted by effect size. The dots represent the ES; the diamonds represent the weighted average ES; the error bars indicate the 95% confidence interval (CI). Studies that used multiple cooling trials were included more than once

Twenty-seven experiments (exercise duration: 33.6 ± 22.8 min) were available for a constant workload exercise performance analysis, of which 16 experiments used a pre-cooling intervention and 11 experiments used a per-cooling intervention. Cold/ice water ingestion (20.0%), cooling vests (19.6%), and menthol use (17.7%) were most frequently adopted as cooling strategies (Fig. 1 of the ESM). The median weighted improvement in constant workload exercise performance was ES = 0.62, 95% CI 0.44–0.81. Pre-cooling was applied prior to time to exhaustion (13 out of 16 experiments), an intermittent exercise (1 out of 16 experiments), or a fixed RPE (2 out of 16 experiments) protocol. Per-cooling was only used during one time-to-exhaustion protocol. Constant workload exercise performance improvements did not differ for pre-cooling (ES = 0.74, 95% CI 0.50–0.98) (Fig. 5) versus per-cooling (ES = 0.45, 95% CI 0.16–0.74 (Fig. 6), *p* = 0.13). Nevertheless, the magnitude of the ES differed across cooling strategies, with no benefits of limb per-cooling (ES =  − 0.18, 95% CI − 0.98 to 0.74) to large benefits of a cooling vest during pre-cooling (ES = 0.81, 95% CI 0.27–1.35) or per-cooling interventions (ES = 1.15, 95% CI 0.30–2.01) (Figs. 5, 6). Statistical heterogeneity was only observed for pre-cooling (*I*^2^ = 62%, *p* < 0.001) studies and not for per-cooling (*I*^2^ = 36%, *p* = 0.11).

### Self-Paced Versus Constant Workload Exercise Studies

**Fig. 7:**
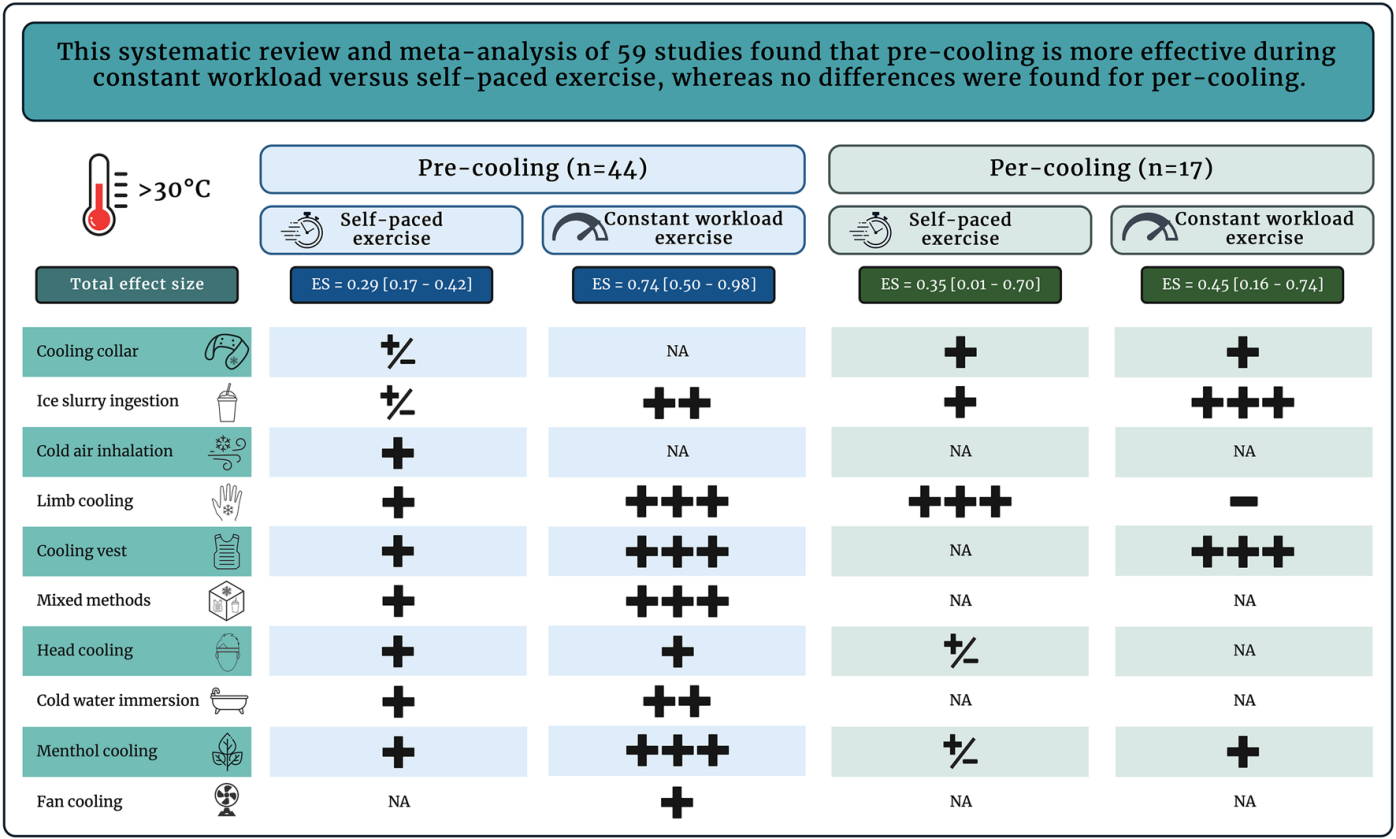
Graphical summary: effectiveness of pre- and per-cooling strategies on performance outcomes of self-paced versus constant workload exercise protocols. Pre-cooling was more effective for constant workload versus self-paced exercise, whereas no differences were found for per-cooling strategies. The effectiveness of different cooling techniques was also explored. The magnitude of the effect was classified as: < 0.0 = negative ( −), 0.0–0.19 = trivial ( ±), 0.2–0.49 = small ( +), 0.5–0.79 = moderate (+ +), and > 0.8 = large (+ + +). *ES* effect size, *NA* not available. Created with BioRender.com

The type of exercise protocol impacted the magnitude of performance benefits following cooling interventions, with a smaller improvement following self-paced versus constant workload exercise (ES = 0.30, 95% CI 0.18–0.42 vs ES = 0.62, 95% CI 0.44–0.81, *p* = 0.004). Interestingly, the difference in performance improvement between self-paced and constant workload exercise was only observed with pre-cooling interventions (ES = 0.29, 95% CI 0.17–0.42 vs ES = 0.74, 95% CI 0.50–0.98, *p* = 0.001, Fig. 2 of the ESM), but not with per-cooling interventions (ES = 0.35, 95% CI 0.01–0.70 vs ES = 0.45, 95% CI 0.16–0.74, *p* = 0.68, Fig. 3 of the ESM). Figure 7 provides a graphical summary of the results. Further stratification for exercise duration revealed that differences in the effectiveness of pre-cooling between self-paced and constant workload studies were larger for exercise protocols with a short-to-medium duration (< 40 min) [ES = 0.26, 95% CI 0.09–0.43 vs ES = 0.90, 95% CI 0.62–1.19, *p* < 0.001, Fig. 4 of the ESM). However, this effect was not present for protocols with a medium-to-long duration (> 20 min) [ES = 0.30, 95% CI 0.16–0.43 vs ES = 0.43, 95% CI 0.13–0.73, *p* = 0.44, Fig. 5 of the ESM) or medium duration only (20–40 min) [ES = 0.25, 95% CI 0.05–0.45 vs ES = 0.47, 95% CI 0.04–0.91, *p* = 0.36, Fig. 6 of the ESM).

## Discussion

The purpose of this meta-analytical review was to compare the magnitude of the performance effect from pre- and per-cooling on self-paced and constant workload exercise performance. For this purpose, data from 40 self-paced and 19 constant workload studies were pooled, representing performance outcomes of 832 paired measurements. We found that pre-cooling provided less performance enhancement during self-paced compared with constant workload exercise in the heat (ES = 0.29, 95% CI 0.17, 0.42 vs ES = 0.74, 95% CI 0.50–0.98), whereas no difference in performance was noted for per-cooling across exercise protocols (ES = 0.35, 95% CI 0.01–0.70 vs ES = 0.45, 95% CI 0.16–0.74). We also observed a large heterogeneity in the benefits of cooling interventions within exercise protocols. These findings have important implications for competitive athletes as the performance benefits of pre-cooling during self-paced exercise may be less than previously assumed.

Cooling interventions did not produce similar performance benefits for self-paced and constant workload exercise in the heat. It was previously suggested that the type of exercise protocol may impact the magnitude of performance benefits [10, 11]. To account for these methodological differences, we calculated Hedges’ *g* rather than a percentage improvement, so this could not explain our findings. Alternatively, the duration of the exercise protocol may have contributed to this finding as previous studies suggested that pre-cooling interventions are predominantly effective for an exercise duration of < 40 min [6, 84]. Indeed, longer protocols (i.e., > 40 min) were more common in self-paced compared with constant workload studies (47% vs 34% of included experiments), but exclusion of these studies did not alter the outcomes of our analysis (Fig. 4 of the ESM). We also observed that the constant workload studies with the largest attenuation of decline in exercise performance (i.e., > 50%) used the shortest exercise protocol (i.e., < 20 min) [65, 76, 78]. Stratified analyses without these studies resolved the statistical significance between self-paced and constant workload exercise protocols (Figs. 5 and 6 of the ESM), but the ES of the effectiveness of pre-cooling remained substantially higher for constant workload studies (ES = 0.25, 95% CI 0.05–0.45 vs ES = 0.47, 95% CI 0.04–0.91). These findings indicate that the performance benefits following pre-cooling in self-paced versus constant workload protocols are mediated by exercise duration, with differences mainly present during shorter exercise protocols.

Other explanations for the observed differences may relate to exercise and intervention characteristics. For example, thermal perception is known to impact exercise performance in the heat [85], whereas the magnitude of this effect may be exercise and intervention dependent. Furthermore, the absolute workload, and thus heat production, is likely higher during self-paced exercise compared with constant workload exercise, which could lead to a greater heat storage and associated increments in core temperature, compared with constant workload exercise in comparable environmental conditions. The adopted cooling interventions may not have been powerful enough to compensate for the high rate of metabolic heat production during self-paced protocols. However, mixed-method cooling was more often applied to self-paced versus constant workload experiments (25.0% vs 5.8%, Fig. 1 of the ESM) and we have previously demonstrated that this type of cooling exerts the strongest cooling and performance effects [1, 5]. The cooling strategy that was used does, therefore, not explain our findings. This also applies to airflow, as limited or no airflow could overestimate the benefits of cooling [86], but no differences in airflow characteristics were found between protocols.

The observation that pre-cooling has different benefits on self-paced compared with constant workload exercise performance has important practical implications. The quantification of pre-cooling specific performance benefits that were proposed in previous meta-analyses [8, 87] cannot be translated to self-paced exercise settings, as this overestimated the true effect due to the inclusion of constant-workload studies. Instead, exercise protocol and cooling intervention-specific estimates, as presented in our meta-analysis, provide a more accurate quantification of cooling-induced performance benefits. It is also important to emphasize that the lower effectiveness of pre-cooling in self-paced exercise trials does not disqualify the intervention by itself. After all, a statistically significant performance benefit (ES = 0.29, 95% CI 0.17–0.42) was found for self-paced exercise protocols when using any pre-cooling intervention prior to exercising in the heat compared with a control condition without cooling. Hence, the use of pre-cooling strategies, such as a mixed-method intervention (ES = 0.33), a cooling vest (ES = 0.27), or ice slurry ingestion (ES = 0.11), does provide a performance benefit during self-paced exercise under heat stress. It is also important to highlight that the magnitude of performance benefits was highly context specific, depending on the exercise protocol and the type of cooling, given the large range in ES across different cooling interventions (Fig. 3). Furthermore, in some sports (e.g., marathon running, long-distance cycling), a hybrid pacing strategy is adopted, with a near to constant-workload approach. For optimal laboratory-to-field translation of the ergogenic effects of cooling on performance, the characteristics of the sport and cooling type need both to be considered.

We also found that per-cooling provided comparable performance benefits for self-paced and constant workload exercise protocols. This finding further reinforces the use of per-cooling strategies during competition, as it remains less often applied compared with pre-cooling owing to challenges with practical implementation and the additional weight of a cooling garment [88, 89]. A recent study [90] described practical pre-, per-, and post-cooling methods for racewalking and rugby competition during the Tokyo 2020 Olympics. In both sports, a combination of per-cooling methods was allowed and could be used by athletes during competition. Furthermore, the combination of pre- and per-cooling interventions may be superior to the effectiveness of the cooling interventions in isolation [5], but this could not be addressed in the present analysis because of the limited number of studies that adopted a combination of pre- and per-cooling.

Participants in the included studies had a *V*O_2max_ of ~ 56 mL kg^−1^ min^−1^. A previous study [91] showed that the VO_2max_ of elite male athletes ranged between 59 and 77 mL kg^−1^ min^−1^. As higher aerobic fitness levels have been associated with better thermoregulatory control [92, 93], elite athletes might experience smaller benefits from cooling interventions than we reported, given that they may better cope with heat. In contrast, it has been shown that 98% of elite athletes experience a performance decrement during exercise in hot and humid versus temperate conditions [94]. These observations underline the potential of pre- and per-cooling as valuable heat mitigation strategies for both amateur and elite athletes.

A major strength of this study is the large number of included experiments (*n* = 86 with 832 paired measurements), as well as the comparison of performance benefits between distinct exercise protocols and the impact of the different pre- and per-cooling interventions on this association. However, some limitations should be considered. First, we excluded 11 studies that combined a constant workload and a self-paced exercise protocol because it was impossible to distinguish the direct effect of cooling on either of the exercise protocols. Second, only data from male individuals were used within this review as very few studies report performance data in female participants. Caution must therefore be used when inferring results from these studies in male individuals directly to female individuals, as female individuals have a limited evaporative capacity at high levels of heat production due to sex-mediated differences in sweat gland output [95]. Given the under-representation of female individuals in exercise science, future studies and a meta-analysis on the benefits of cooling interventions on performance benefits of female athletes during exercise in the heat are warranted. Finally, insufficient data were available to perform stratified analyses for cooling type, cooling dose, exercise type, and training status, thus future meta-analyses should take this into account.

## Conclusions

Cooling interventions attenuate the decline in performance during exercise in the heat, but the magnitude of the effect is dependent on the exercise protocol (self-paced vs constant workload) and type of cooling (pre- vs per-cooling). Pre-cooling appears to be more effective during a constant workload compared with self-paced exercise protocols, whereas no differences were found in the effectiveness of per-cooling. We also observed substantial heterogeneity in the magnitude of performance benefits across different type of cooling interventions, thus additional studies regarding which type of cooling is most effective under specific exercise conditions (e.g., type, duration) are warranted.

### Supplementary Information

Below is the link to the electronic supplementary material.

Supplementary file1 (PDF 1363 kb)

## Declarations

### Availability of Data and Material

The dataset that was used for the meta-analysis is available upon reasonable request to the corresponding author.
