# Supplementary material for: Performance Benefits of Pre- and Per-cooling on Self-paced Versus Constant Workload Exercise: A Systematic Review and Meta-analysis
Source: Sports Med. 2023 Oct 6;54(2):447–71. doi: 10.1007/s40279-023-01940-y (PMC10933154; doi:10.1007/s40279-023-01940-y)
Supplement: Supplementary file 1 — Supplementary file1 (PDF 1363 kb) [file 40279_2023_1940_MOESM1_ESM.pdf]

Supplemental file 1.

To:

Performance benefits of pre- and per-cooling on self-paced *versus* constant workload exercise: a systematic review and meta-analysis

Running title: Pre- and per-cooling on self-paced *versus* constant workload exercise

Tessa M. van de Kerkhof[1] – ORCID: 0000-0001-6339-1633

Coen C.W.G. Bongers[1,2] – ORCID: 0000-0003-0055-5308

Julien D. Périard[3]– ORCID: 0000-0002-6266-4246

Thijs M.H. Eijsvogels[1] – ORCID: 0000-0003-0747-4471

Affiliations:

[1] Radboud university medical center, Radboud Institute for Health Sciences, Department of Physiology, Nijmegen, The Netherlands.

[2] HAN university of Applied Sciences, Health Promotion and Performance, Nijmegen, The Netherlands.

[3] University of Canberra Research Institute for Sport and Exercise, Canberra, Australia

Address for correspondence:

Dr. Thijs Eijsvogels, Department of Physiology (392), Radboud university medical center, P.O. Box 9101, 6500 HB Nijmegen, The Netherlands, Tel +31 24 36 13 674, Fax +31 24 36 16413,

E-mail: Thijs.Eijsvogels@radboudumc.nl

Supplemental table 1. Search strategy

Theme	Pubmed
Exercise	("exercise"[MeSH Terms] OR "exercis*"[tiab] OR "sports"[MeSH Terms] OR "sport*"[tiab] OR cycling [tiab] OR bicycl*[tiab] OR running[tiab])
Cooling interventions	("cooled"[Tiab] OR "cooling"[Tiab] OR "coolings"[Tiab] OR "cools"[Tiab] OR "cooled"[Tiab] OR "cool"[Tiab] OR "precool*"[Tiab] OR "pre-cool*"[Tiab] OR "midcool*"[Tiab] OR "per-cool*"[Tiab] OR "percool*"[Tiab] OR ((cold[tiab] OR ice[tiab]) AND (water[tiab] OR drink[Tiab] OR slurry[tiab] OR fluid[Tiab] OR slush*[tiab]) AND (ingest*[tiab] OR immersion[tiab] OR immersed[tiab] OR intake[tiab])) OR (facial[tiab] AND water[tiab] AND spray[tiab]) OR "cold pack*"[Tiab] OR "menthol"[MeSH Terms] OR "menthol"[Tiab])
Exercise performance	(athletic performance [MeSH Terms] OR "perform*"[tiab] OR “physical endurance” [MeSH Terms] OR ((time[tiab] OR duration [tiab]) AND (exhaustion[tiab] OR finish[tiab] OR exercise[tiab])) OR ((mean[tiab] OR total[tiab]) AND (“power output”[tiab] OR work[tiab] OR distance[tiab])))

Theme	Web of Science (*WoS categories: sport sciences, physiology, medicine general internal, nutrition dietetics, orthopedics, rehabilitation, cardiac cardiovascular systems, medicine research experimental)*
Exercise	("exercis*" OR "sport*" OR cycling OR bicycl* OR running)
Cooling interventions	("cooled" OR "cooling" OR "coolings" OR "cools" OR "cooled" OR "cool" OR "precool*" OR "pre-cool*" OR "midcool*" OR "per-cool*" OR "percool*" OR ((cold OR ice) AND (water OR drink OR slurry OR fluid OR slush*) AND (ingest* OR immersion OR immersed OR intake)) OR (facial AND water AND spray) OR (facial AND water AND spray) OR "cold pack*" OR "menthol")
Exercise performance	(“athletic performance” OR "perform*" OR “physical endurance” OR ((time OR duration) AND (exhaustion OR finish OR exercise)) OR ((mean OR total) AND (“power output” OR work OR distance)))

Theme	Medline (*Multi-field search, only search in abstract)*
Exercise	(exercis* OR sport* OR cycling OR bicycl* OR running)
Cooling interventions	(cooled OR cooling OR coolings OR cools OR cooled OR cool OR precool* OR pre-cool* OR midcool* OR per-cool* OR percool* OR ((cold OR ice) AND (water OR drink OR slurry OR fluid OR slush*) AND (ingest* OR immersion OR immersed OR intake)) OR (facial AND water AND spray) OR (facial AND water AND spray) OR cold pack* OR menthol)
Exercise performance	(athletic performance OR perform* OR physical endurance OR ((time OR duration) AND (exhaustion OR finish OR exercise)) OR ((mean OR total) AND (power output OR work OR distance))

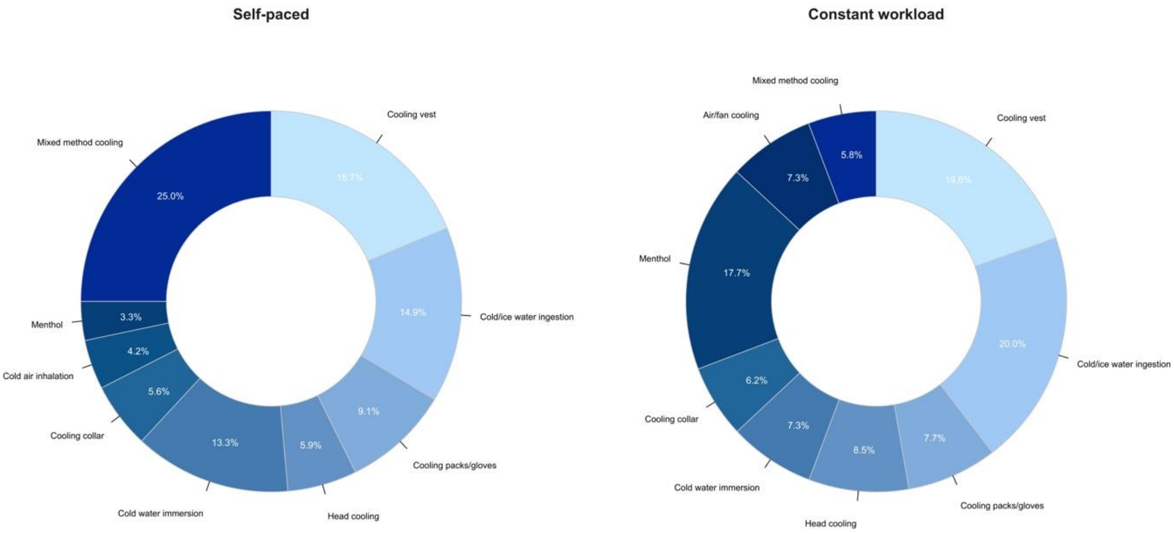

Supplemental figure 1. Pie charts showing percentage use of cooling interventions for self-paced (left) and constant workload (right) studies.

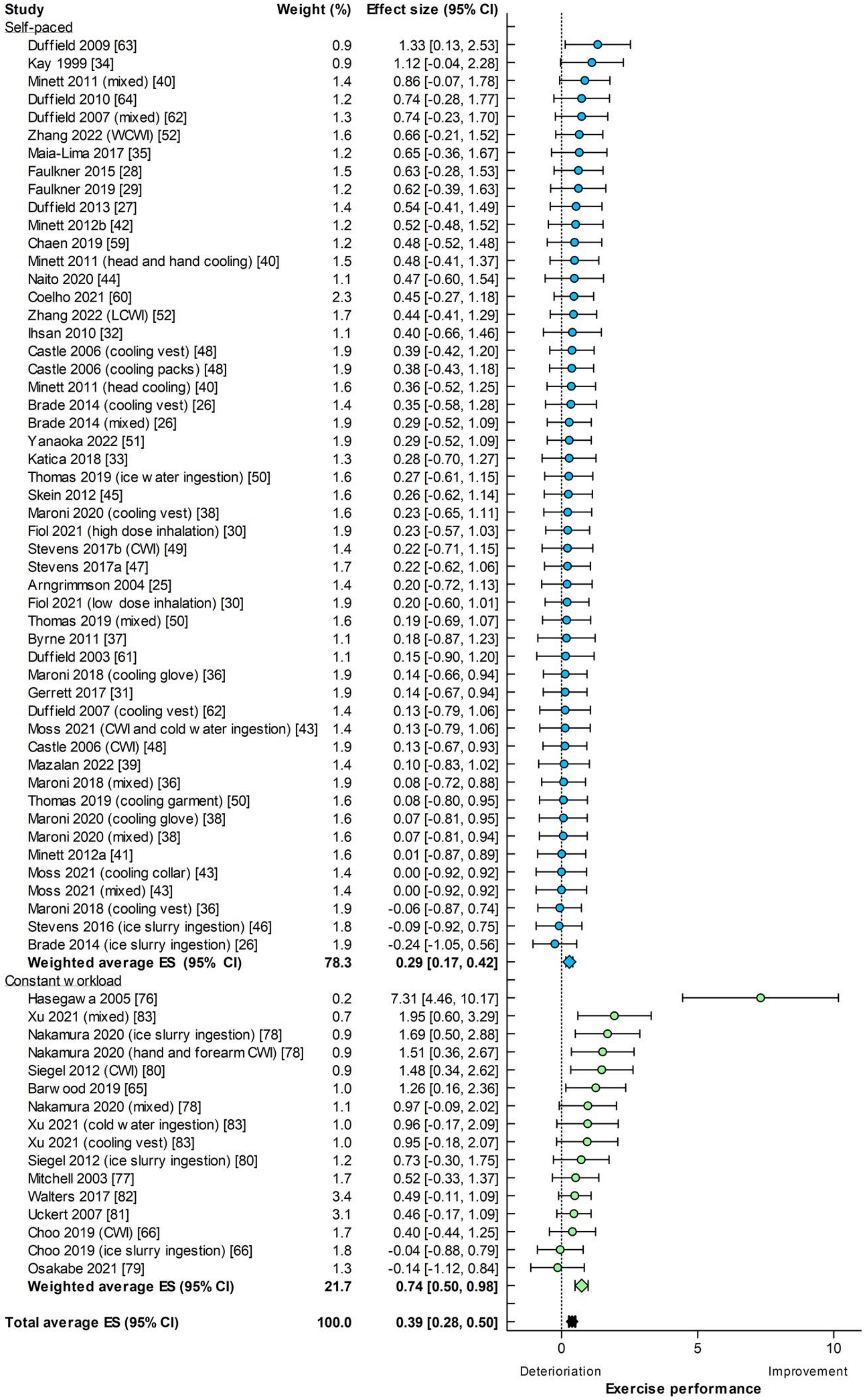

Supplemental figure 2. Forest plot summarizing the effect of pre-cooling on exercise performance (ES in Hedge’s g) for the self-paced (light blue) and constant workload (light green) studies separately. The dots represent the ES; the diamonds represent the weighted average ES; the error bars indicate the 95% CI. Studies that used multiple cooling trials were included more than once.

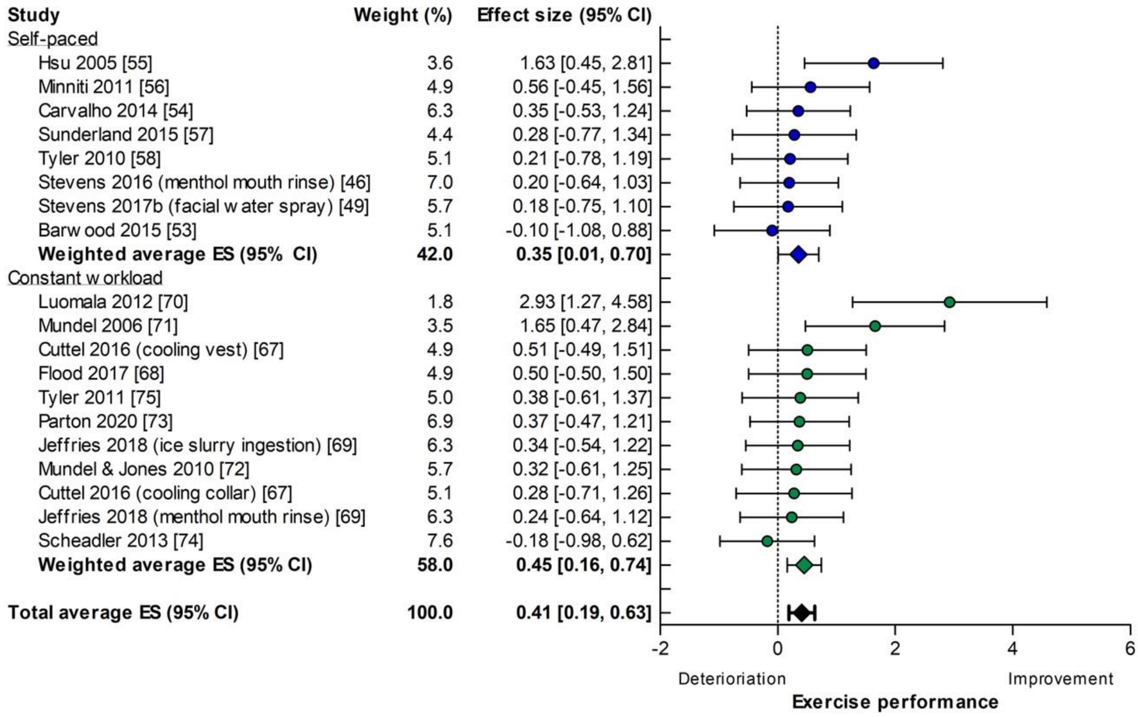

Supplemental figure 3. Forest plot summarizing the effect of per-cooling on exercise performance (ES in Hedge's g) for the self-paced (dark blue) and constant workload (dark green) studies separately. The dots represent the ES; the diamonds represent the weighted average ES; the error bars indicate the 95% CI. Studies that used multiple cooling trials were included more than once.

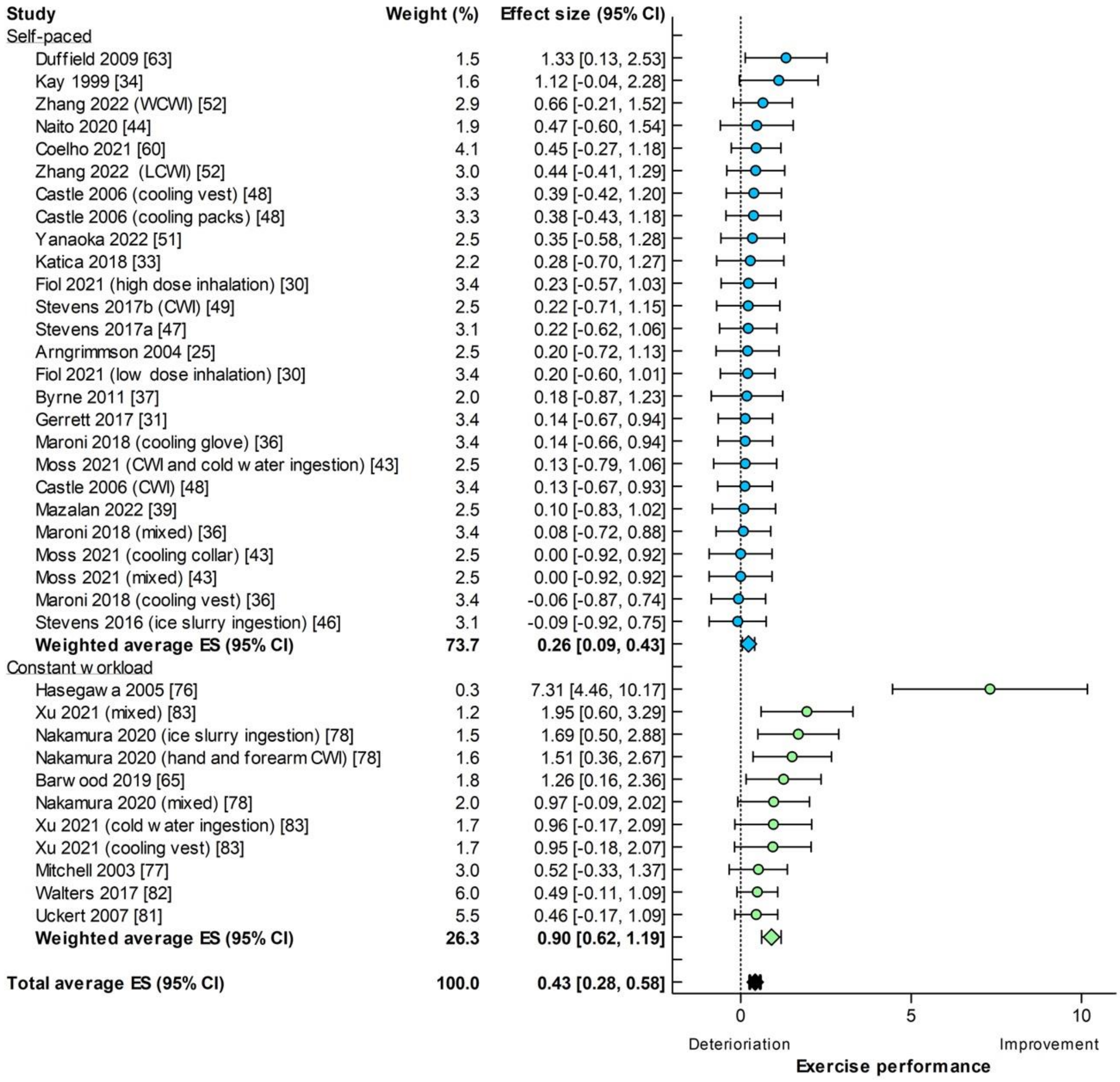

Supplemental figure 4. Forest plot summarizing the effect of pre-cooling on exercise performance (ES in Hedge's g) for the self-paced (light blue) and constant workload (light green) studies that used an exercise protocol <40 minutes. The dots represent the ES; the diamonds represent the weighted average ES; the error bars indicate the 95% CI. Studies that used multiple cooling trials were included more than once.

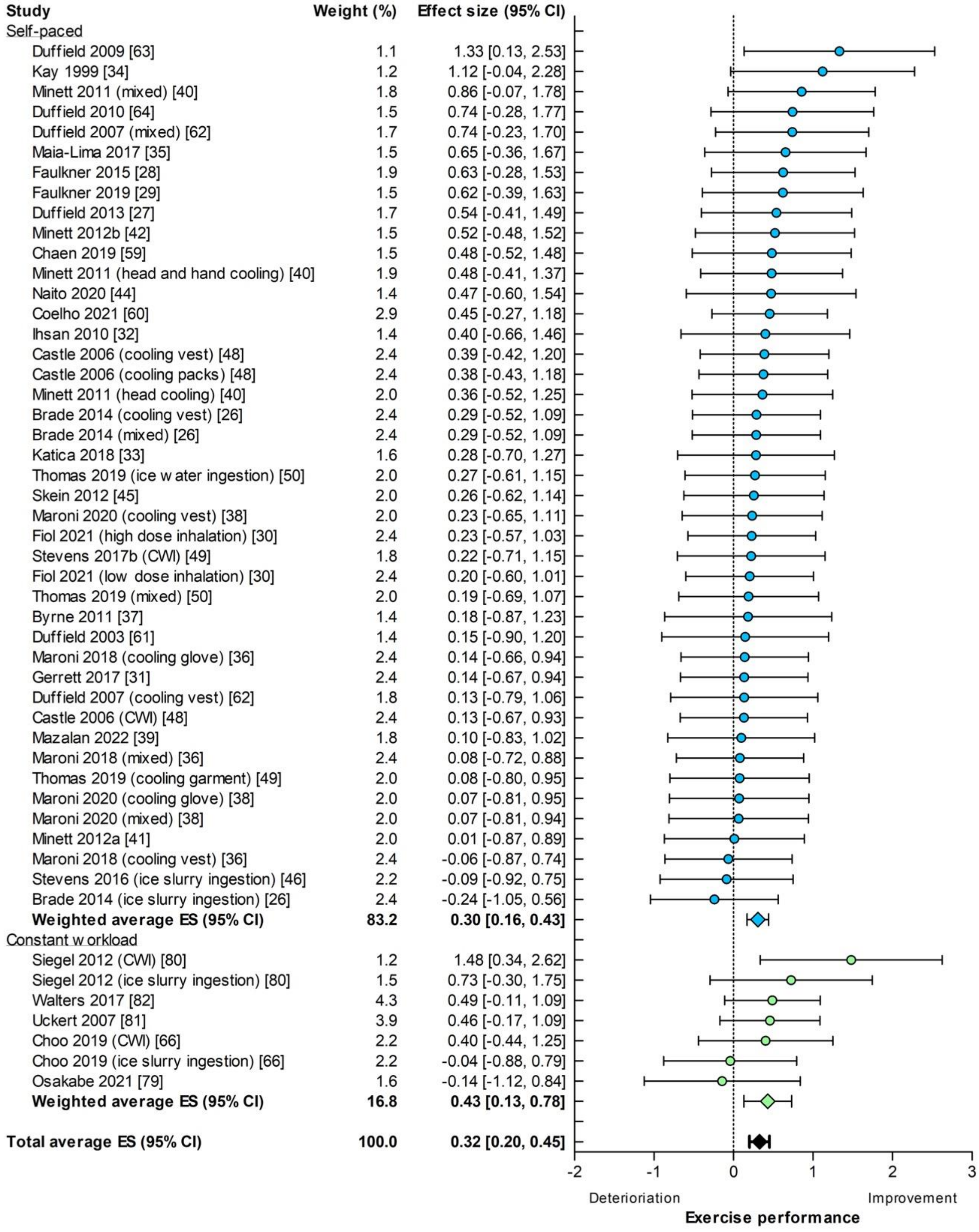

Supplemental figure 5. Forest plot summarizing the effect of pre-cooling on exercise performance (ES in Hedge's g) for the self-paced (light blue) and constant workload (light green) studies that used an exercise protocol >20 minutes. The dots represent the ES; the diamonds represent the weighted average ES; the error bars indicate the 95% CI. Studies that used multiple cooling trials were included more than once.

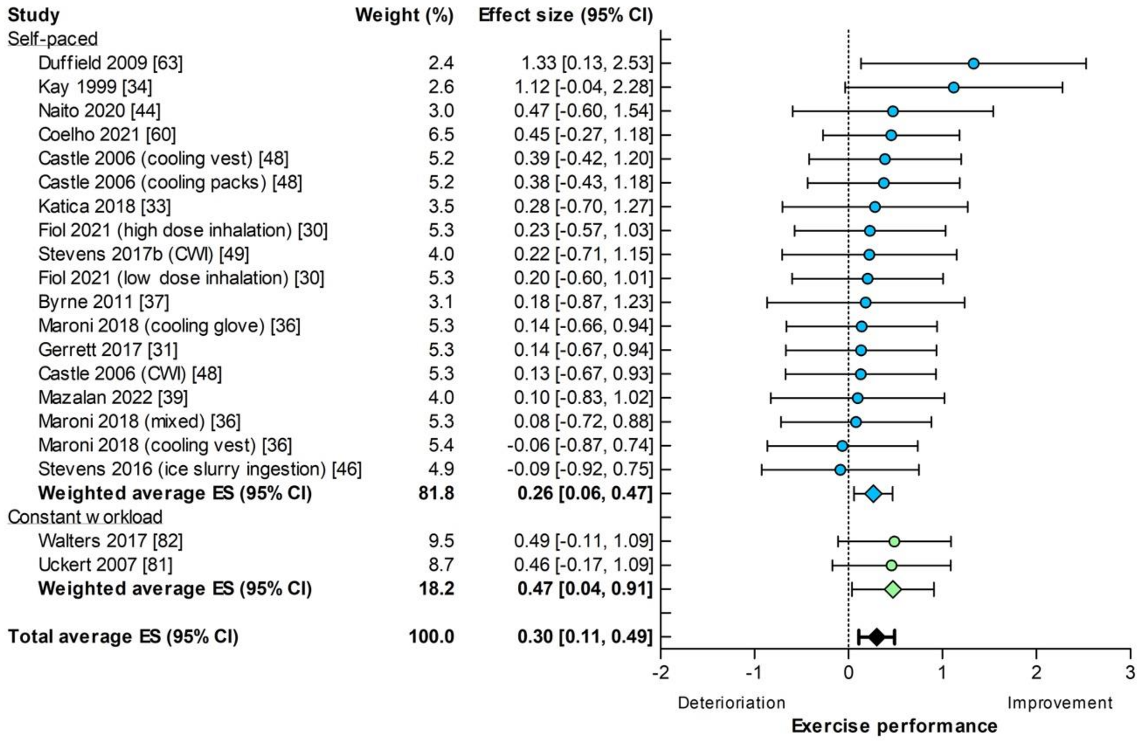

Supplemental figure 6. Forest plot summarizing the effect of pre-cooling on exercise performance (ES in Hedge's g) for the self-paced (light blue) and constant workload (light green) studies that used an exercise protocol 20-40 minutes. The dots represent the ES; the diamonds represent the weighted average ES; the error bars indicate the 95% CI. Studies that used multiple cooling trials were included more than once.
